# *In vitro *antimicrobial susceptibility of fosfomycin against organisms isolated from various clinical specimens: a multicentre trial from Kolkata

**DOI:** 10.1186/cc14176

**Published:** 2015-03-16

**Authors:** A Chakraborty, S Roy, S Chakraborty, A Datta, A Kar

**Affiliations:** 1 Medica Superspecialty Hospital, Kolkata, India

## Introduction

In the era of rising prevalence of serious infections caused by multidrug-resistant (MDR) organisms and the paucity of in-flow of newer antimicrobial agents, the relatively older antibiotics that had been left out of clinical practice for various reasons are now being increasingly considered as the potential agents to combat such infections. Fosfomycin, known for almost four decades, has a broad spectrum of activity against several Gram-negative and Gram-positive bacteria.

## Methods

This study, conducted in the Microbiology Department of Medica Superspecialty Hospital between July and November 2014, was aimed at testing the *in vitro *sensitivity of fosfomycin against isolates identified from various clinical specimens from different parts of Kolkata. After confirming the identity and antibiogram by Microscan Autoscan 4, the isolates were tested for fosfomycin sensitivity by the Epsilometer test. MIC values were interpreted in accordance with the currently recommended Clinical and Laboratory Standards Institute (CLSI) criteria for urinary tract isolates of *Escherichia coli *and *Enterococcus faecalis *and the European Committee on Antimicrobial Susceptibility Testing (EUCAST) criteria for Enterobacteriaceae and *Staphylococcus aureus*.

## Results

Out of the 1,895 isolates tested, fosfomycin displayed an overall *in vitro *susceptibility against 90%, but only 64% against MDR strains. Among the MDR organisms nearly 78% of *E. coli *and 70% of *Klebsiella *spp. and 40% of MRSA isolates showed provisional MICs in the sensitive range while among the sensitive strains fosfomycin showed around 92% susceptibility. Our study results were comparable with the results obtained from an Indian study published from CMC Vellore in 2013 showing a fosfomycin susceptibility of around 75% among MDR uropathogenic *E. coli*.

## Conclusion

Being a broad-spectrum bactericidal agent usable both orally and parenterally with low toxicity profiles and lesser prevalence of cross-resistance with other antimicrobials, fosfomycin can be an alternative to other broad-spectrum agents to treat uncomplicated infections as well as in the case of infections with MDR organisms where treatment options are very few. This study possibly reveals a much-needed solution for the rising carbapenem resistance and also for the treatment of infections with such MDR pathogens, thereby bringing down the length of stay in hospital, cost of therapy and suffering on the part of the patients.

